# Generative Modeling of InSAR Interferograms

**DOI:** 10.1029/2018EA000533

**Published:** 2019-12-21

**Authors:** Guillaume Rongier, Cody Rude, Thomas Herring, Victor Pankratius

**Affiliations:** ^1^ Department of Earth, Atmospheric, and Planetary Sciences Massachusetts Institute of Technology Cambridge MA USA; ^2^ Kavli Institute for Astrophysics and Space Research Massachusetts Institute of Technology Cambridge MA USA

**Keywords:** InSAR, surface deformation, generator, machine learning, geostatistics

## Abstract

Interferometric synthetic aperture radar (InSAR) has become an essential technique to detect surface variations due to volcanoes, earthquakes, landslides, glaciers, and aquifers. However, Earth's ionosphere, atmosphere, vegetation, surface runoff, etc., introduce noise that requires post‐processing to separate its components. This work defines a generator to create interferograms that include each of those components. Our approach leverages deformation models with real data, either directly or through machine learning using geostatistical methods. These methods result from previous developments to more efficiently and better simulate spatial variables and could replace some statistical approaches used in InSAR processing. We illustrate the use of the generator to simulate an artificial interferogram based on the 2015 Illapel earthquake and discuss the improved performance offered by geostatistical approaches compared with classical statistical ones. The generator establishes a tool for multiple applications (1) to evaluate InSAR correction workflows in controlled scenarios with known ground truth; (2) to develop training sets and generative methods for machine learning algorithms; and (3) to educate on InSAR and its principles.

## Key Points

- We introduce a software tool that can generate artificial interferograms for synthetic aperture radar (SAR) applications
- The tool leverages real data and geostatistical methods to generate and perturb interferogram components
- It can be used to evaluate InSAR error correction workflows, to enhance machine learning use with InSAR, and to teach InSAR principles

## 1 Principles and Techniques of Synthetic Aperture Radar Interferometry

**Figure 1:**
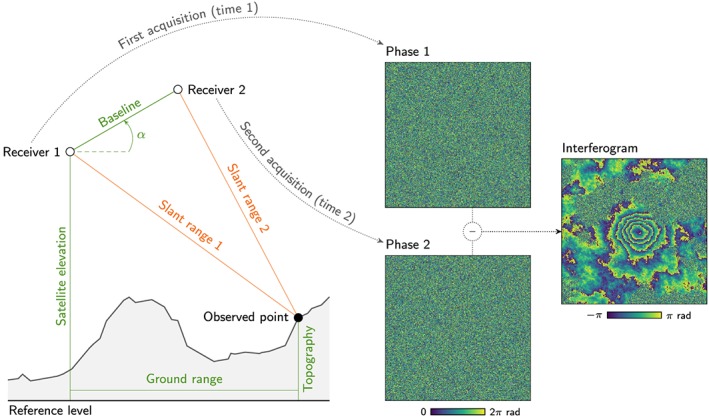
Acquisition scene reproduced by our InSAR interferogram generator. Receivers 1 and 2 can be configured as either two receivers on a single satellite measuring simultaneously or a single receiver measuring during two passes at two different times. Input parameters for the generator are shown in green; other input parameters (not shown) include the satellite track angle and look direction. The slant range outputs shown in orange are used to generate the interferogram.

Interferometric synthetic aperture radar (InSAR) is extensively used in geosciences to create digital elevation models and to map Earth surface deformations. Creating an interferogram relies on measuring the phase difference between two signals sent from different orbital positions looking at the same point on Earth (Moreira et al., 2013; Figure 1). InSAR satellites operate in the microwave frequency range, which enables ground observations in the presence of clouds and during the night.

In practice, the reflected signal *ϕ* is a superposition of several components, which can be attributed to different phenomena (Hanssen, 2001; Moreira et al., 2013):

(1) $$\phi =\phi_{\text{look}}+\phi_{\text{flat}}+\phi_{\text{topo}}+\phi_{\text{defo}}+\phi_{\text{atmo}}+\phi_{\text{temp}}+\phi_{\text{noise}},$$

with contributions due to *ϕ*_look_ satellite angle of observation; *ϕ*_flat_ spheroidal shape of the Earth; *ϕ*_topo_ topography; *ϕ*_defo_ surface deformation; *ϕ*_atmo_ atmospheric delay; *ϕ*_temp_ temporal decorrelations, for example, due to rockslides, sediment transport, and vegetation changes; and *ϕ*_noise_ remaining perturbations, for example, due to thermal and orbital errors.

Among these components, the topography *ϕ*_topo_ and the surface deformation *ϕ*_defo_ draw the most interest. However, their extraction is challenging. A major difficulty is that components such as atmospheric delays *ϕ*_atmo_ can be an order of magnitude larger than the other signals and mask the surface deformations (Bekaert et al., 2015), which means that any improvements in quantitative estimation can have a big practical impact.

Current methods focus on modeling the atmospheric delays using empirical relationships, data extracted from GPS signals, data extracted from satellites' spectrometers, and/or atmospheric models, sometimes adding another layer of processing using statistical models (e.g.,Bekaert et al., 2015; Sudhaus & Sigurjón, 2009; Wright et al., 2003). Such methods typically give fewer attention to other sources of component perturbation, such as temporal decorrelation. And few studies have quantified the impact of all perturbations and the related processing errors on the estimation of the topography or on the estimation of the deformation sources (e.g., Dawson & Tregoning, 2007; Scott & Lohman, 2016).

To tackle these problems, this work creates an open‐source software tool with several applications in mind. It mimics the real InSAR acquisition process, so it provides a controlled environment to evaluate InSAR error correction algorithms and workflows in a systematic way and can help teach InSAR principles. In addition, it can generate hypothetical interferograms of what would be expected under certain conditions for a given region. This synthesis functionality is meant to provide a foundation for future applications of generative machine learning algorithms in InSAR error correction.

We outline the underlying model of the generator in section 2. Section 3 presents an illustrative application to the 2015 Illapel Earthquake, while section 4 discusses possible extensions.

## 2 Generation of Artificial Interferograms

### 2.1 Slant Range and Phase Computation

Our generative approach relies on the simulation of an InSAR acquisition scene (Figure 1). The satellite track angle (i.e., azimuth of the satellite's path), the look direction (i.e., left or right), and the initial position of the first receiver when the acquisition starts determine the satellite position during the observation for each point of the simulation domain (see Appendix A).

Initially, the generator requires the minimal ground range between the first receiver and the closest observed point and the satellite's altitude. These values determine the initial position of the first receiver based on the ground range, look direction, and coordinates of the closest observed point. Subsequent orbital values of the first and second receivers at a known baseline distance and angle *α* can be derived as shown in Appendix A.

The slant range $r_{i,j}^{s}$ results from the Euclidean distance between the coordinates of each observed point $\left(x_{i}^{o},y_{i}^{o},z_{i}^{o}\right)$, *i*=1…*n*, and of a receiver $\left(x_{i,j}^{r},y_{i,j}^{r},z_{i,j}^{r}\right)$, *j*=1 or 2:

(2) $$r_{i,j}^{s}=\sqrt{{\left(x_{i,j}^{r}-x_{i}^{o}\right)}^{2}+{\left(y_{i,j}^{r}-y_{i}^{o}\right)}^{2}+{\left(z_{i,j}^{r}-z_{i}^{o}\right)}^{2}}.$$

The unwrapped phase *ψ*_*i*,*j*_ is derived from the slant range and the wavelength *λ* of the radar signal (Hanssen, 2001):

(3) $$\psi_{i,j}=-\frac{2k\pi }{\lambda }(r_{i,j}^{s}),$$

with *k*=1 for one‐way ranging and *k*=2 for two‐way ranging. The wrapped phase $\psi_{i,j}^{w}$, typically defined on [0,2*π*), is given by

(4) $$\psi_{i,j}^{w}=\text{mod}(\varphi_{i,j},2\pi ).$$

Finally, the unwrapped interferometric phase *ϕ*_*i*_ is expressed as follows:

(5) $$\phi_{i}=\psi_{i,1}-\psi_{i,2},$$

which defines the wrapped interferometric phase $\phi_{i}^{w}$ on [−*π*,*π*) as follows:

(6) $$\begin{array}{ccc} \phi_{i}^{w} & =\text{mod}(\phi_{i}+\pi ,2\pi )-\pi & \\ & =\text{mod}((\psi_{i,1}^{w}-\psi_{i,2}^{w})+\pi ,2\pi )-\pi . \end{array}$$

In a real case, $\psi_{i,j}^{w}$ is obtained after processing the satellite raw data, and the second equality in equation (6) gives the interferogram. As such, the generator is able to reproduce any satellite configuration, whether it uses L, C, or any other band and 1, 2, or more passes.

### 2.2 Real Topography Infusion

The simulation domain of the generator is defined by a Cartesian grid, and the center of each cell represents an observed point on Earth. The simulation requires the coordinates and elevation (i.e., the topography) of each point.

We use elevation data from the Shuttle Radar Topography Mission (SRTM; Farr et al., 2007) instead of simulating artificial topographies. SRTM data cover most of Earth's land masses at three arc seconds (about 90 m) and one arc second (about 30 m). Our framework accesses them through the *Scikit Data Access* Python API (Rude et al., 2017a). SRTM data contain the elevation above the EGM96 geoid, which has to be converted to elevation above an ellipsoid when using a spherical or ellipsoidal reference level (see Appendix A).

### 2.3 Surface Deformation Modeling

Surface deformation is often the key signal of interest in an interferogram. They can result from many processes, for instance, load changes at the surface or ruptures and pressure changes in the subsurface.

Deformation processes are complex and depend on multiple parameters, such as topography, gravity, underground geological materials, and vertical and horizontal heterogeneity of those materials. The vast majority of InSAR studies (e.g.,Lu & Dzurisin, 2014; Scott & Lohman, 2016; Sudhaus & Sigurjón, 2009) relies on approximate models of the source embedded in an elastic half‐space where Earth's crust is represented as a semi‐infinite, isotropic, and linearly elastic space with a flat surface as an upper boundary.

A variety of models exist to simulate the surface deformation from different sources in an elastic half‐space (e.g., Dzurisin, 2006). Our approach implements the following five most common ones in the open‐source Python package *PyInSAR*:

- A uniform disk load (Farrell, 1972), for example, following a magmatic eruption.
- A spheroidal pressure source (also called Mogi source; Mogi, 1958), for example, a magma chamber.
- A closed‐conduit pressure source (Bonaccorso & Davis, 1999), for example, a highly elongated magma chamber or a blocked conduit between a chamber and the surface.
- An open‐conduit pressure source (Bonaccorso & Davis, 1999), for example, an open conduit between a magma chamber and the surface.
- A rectangular dislocation source (also called Okada's model; Okada, 1985), for example, a fault or a dike.

Illustrations of those models are available in the Jupyter notebook /blob/master/geo/insar/simulator/DInSAR_Simulator_Deformation_Models.ipynb at Rude et al. (2017b) and Thomas et al. (2016).

### 2.4 Noise and Scene Perturbation

The implementation so far can model *ϕ*_look_, *ϕ*_flat_, *ϕ*_topo_, and *ϕ*_defo_. This section introduces the three components left from equation (1): *ϕ*_atmo_, *ϕ*_temp_, and *ϕ*_noise_.

All those perturbations are not just random noise: They contain a varying level of spatial correlation. Thus, our generator leverages geostatistical methods to stochastically simulate the noise and perturbations that affect an interferogram.

We focus on two methods: the sequential Gaussian simulation (SGS) and the multiple‐point simulation. Those methods use the sequential simulation approach (Deutsch & Journel, 1992), which randomly iterates over the domain's grid to simulate a value for each cell based on data and previously simulated values and on a prior model. This prior model defines the distribution and spatial patterns of the variable to simulate. Different simulation methods can use different types of prior models, but the principle remains the same: reproducing in the realizations the statistical properties of the prior model.

The SGS (Deutsch & Journel, 1992) belongs to a category of machine learning methods called Gaussian processes (Rasmussen & Williams, 2006). Its prior model includes a histogram that describes the distribution of the variable to simulate and a function that describes the spatial correlation of the variable. This function is usually a variogram in geostatistics and a covariance function (also called kernel) in machine learning. Being a machine learning method, the prior model can be learned from training data, for instance, from interferograms where one component has been separated or is large enough to hide the others. In this case, a model of variogram is fitted to an experimental variogram computed from the data. Variograms require few parameters, and a model can be easily defined manually based on prior knowledge of the phenomenon to simulate. Thus, the SGS is especially suitable when training sets are unavailable or sparse. However, it is limited in its ability to simulate complex spatial patterns (Journel, 2005).

Multiple‐point simulation has been developed to overcome SGS' limitations. Its prior model is not a function of spatial correlation anymore but an image representing the patterns to simulate, called a training image (Guardiano & Srivastava, 1993). Our framework implements a specific multiple‐point simulation method, the direct sampling (DS; Mariethoz te al., 2010a). For each cell to simulate, the DS measures the distance between the pattern of neighboring cells with known values and the patterns in the training image with the same configuration. The simulated value becomes that of the pattern with the smallest distance value. To avoid going through the entire training image, a distance threshold is used: Once a pattern with a distance value smaller than the threshold is found, this pattern provides the simulated value. The DS is able to reproduce complex spatial patterns (Mariethoz et al., 2010a; Meerschman et al., 2013). However, it requires a fully known image of the variable to simulate and can be computationally demanding.

Based on SGS or DS, the generator simulates three types of perturbations: perturbations of the orbital parameters, temporal decorrelations, and atmospheric delays.

Orbital parameters often vary slightly along the satellite's path, perturbing the interferograms. We generate such perturbation using a one‐dimensional SGS or DS, corresponding to a parameter's variations along the satellite path, that we replicate perpendicularly to the satellite path to obtain a value for each cell in the simulation domain. All parameters describing the satellite's position include these uncertainties, that is, track angle, satellite's elevation, minimal ground range, baseline, and *α*.

Temporal decorrelations lead to a loss of coherence between two acquisitions because of small‐scale perturbations, for example, leaf fall, harvesting, and rock falls. Decorrelations vary depending on properties such as the terrain materials, the slope, or the type of vegetation. The generator uses such variations to simulate areas with different temporal decorrelations.

Atmospheric delays correspond to a superposition of several components. The main signal results from tropospheric perturbations due to variations of water vapor, pressure, and temperature. These perturbations include a turbulent component stochastic in space and time and a topography‐correlated component. We account for this dichotomy by using secondary variables in the SGS or DS (Babak, 2009a; Babak & Deutsch, 2009b; Mariethoz et al., 2012). These variables do not provide explicit values of the variable to simulate but influence its spatial continuity. In our case, the secondary variables can account for the topography, large‐scale atmospheric trends, or the slant range without delays, which would induce longer delays as the signal travels longer distances into the atmosphere. Other sources of perturbations such as ionospheric delays could be accounted for, either as secondary variables or as new components to simulate.

## 3 Illustrative Application of Generating a Plausible Interferogram

**Figure 2:**
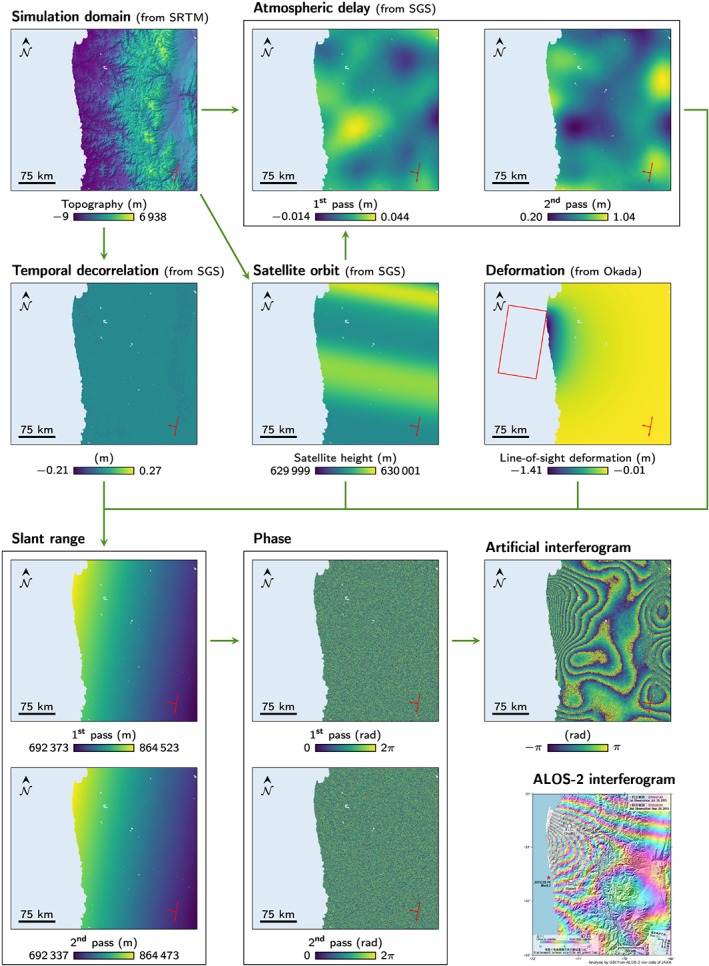
Steps to generate an interferogram including surface deformation, atmospheric delay, and temporal decorrelation. ALOS‐2's interferogram comes from Geospatial Information Authority of Japan (2015). The long and short red arrows indicate the track and look directions of the satellite; the red rectangle represents the fault model projected at the surface. SGS = sequential Gaussian simulation.

This application illustrates the steps to simulate an artificial interferogram using our generator (Figure 2). The interferogram is built on the model of the 2015 Illapel earthquake recorded by the Japanese Advanced Land Observing Satellite 2 (ALOS‐2; Geospatial Information Authority of Japan, 2015). On 16 September 2015, the town of Illapel, Chile, was struck by a magnitude 8.3 earthquake. This earthquake occurred 48‐km west of the city, due to thrust faulting on the subduction zone where the oceanic Nazca plate plunges under the continental South American plate (Hayes et al., 2017). The resulting interferogram (Figure 2, lower right) is a product of ALOS‐2 L‐band radar (wavelength of about 23 cm) with a first pass on 30 July 2015 and a second pass on 24 September 2015; both satellite orbits were along a descending path. The application and related parameters are available in the Jupyter notebook /blob/master/geo/insar/simulator/DInSAR_Simulator_Illapel_Earthquake.ipynb at Rude et al. (2017b).

The topography surrounding Illapel defines the simulation domain: Each cell corresponds to an observed point that requires the generation of an interferometric phase value. The generator first projects the topography into a Lambert azimuthal equal‐area reference frame using the Geospatial Data Abstraction Library (GDAL Development Team, 2015). We use a regular grid spacing of 60 m. The regular structure speeds up the SGS by allowing the pre‐computation of the variogram values, rather than having to recompute them for every simulated cell.

For simplicity, we model the surface deformations due to the earthquake based on the rupture of a single rectangular plane using Okada's model (Okada, 1985). Several studies inverted various data to find the parameters of Okada's model for this earthquake, with different results (Lay et al., 2016; Solaro et al., 2015). Our parameters are based on those studies and a visual comparison with ALOS‐2's interferogram.

The orbital parameters are based on ALOS‐2 orbital specifications (ESA, 2018). These parameters are perturbed using SGS (Figure 2 shows the satellite height as an example). All the parameters that define the prior models of the SGS are defined manually, rather than relying on a predefined model or real data of ALOS‐2 orbital variations. This output is used to compute the satellite's coordinates for each pass, based on a projected Earth model (Appendix A1), and the slant ranges for each pass.

The next steps consist in generating the two most significant sources of perturbations in InSAR: the atmospheric delay and the temporal decorrelation. We use the SGS to generate both of them. The prior models, which determine the distribution and spatial continuity of the perturbations, are estimated manually following two goals: (i) producing features similar to those in ALOS‐2's interferogram and (ii) illustrating what can be done with our framework. Manually defining those parameters enables the exploration of extreme or yet unseen cases. They can also be inferred from interferograms over the same area during a period without significant deformation.

Atmospheric delays are influenced by the topography, and to a lesser degree by the slant range, which changes the time spent by the signal in the atmosphere. The SGS can use those constraints as secondary variables to influence the generated delay. We estimate the slant range for the first pass under the influence of the look angle and the topography only. This slant range and the topography are combined into a single variable, used as secondary data to simulate the atmospheric delays. We simulate one delay for each pass using the SGS (Figure 2).

Temporal decorrelations are simulated for the second pass using the SGS too (Figure 2). Since they are usually not uniform over such a large area, we partition the simulation domain into two classes:

- Areas with a slope lower than 5°, corresponding to the bottom of valleys and the coastal areas. We assign a high decorrelation to such areas with a normal distribution. High decorrelation could occur due to surface runoff and human activity.
- Areas with a slope higher than 5°, which are mainly due to mountain sides. We assign a lower decorrelation to such areas with an exponential distribution. The overall decorrelation in the second class is more spatially limited than in the first class, corresponding to local rock falls and changes in vegetation.

Those two classes are not based on actual data and are just meant to illustrate the simulation of varying spatial characteristics.

Nest, we add the atmospheric delay for the first pass to the slant range of the first pass and the atmospheric delay for the second pass and the temporal decorrelation to the slant range of the second pass. The slant ranges now contain all the *ϕ* components, which represents the signal that would be provided by a satellite (Figure 2). The difference of the phases yields the final interferogram.

## 4 Discussion

**Figure 3:**
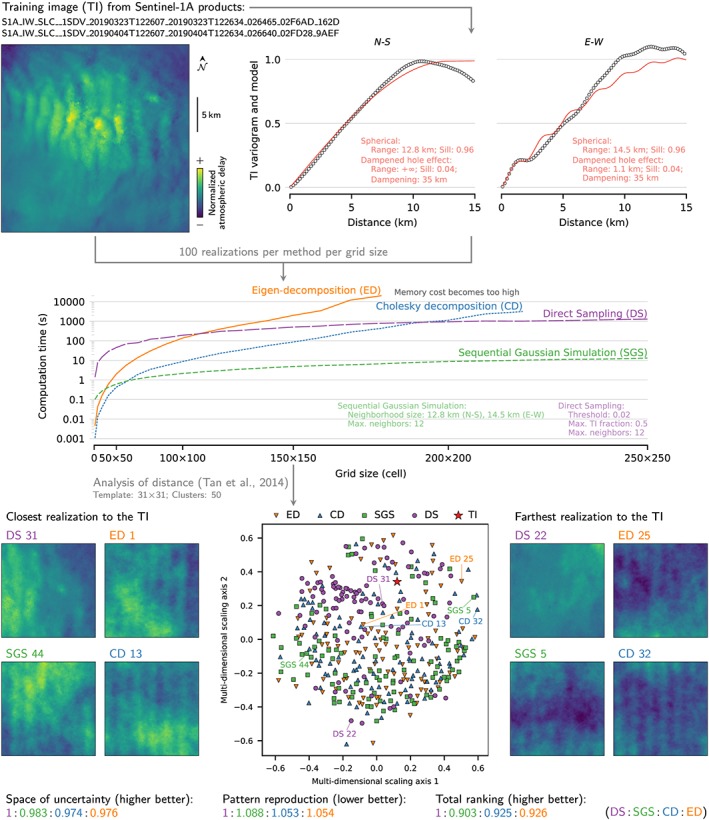
Performance comparison between four methods to simulate random spatial variables on a MacBook Pro with 2.3 Ghz, four‐core Intel Core i5 processor, and 8‐GB memory. Direct sampling uses the training image, while sequential Gaussian simulation, eigen, and Cholesky decompositions use the variogram model. The analysis of distance (Tan et al., 2014) compares pattern frequencies between realizations, capturing multiple‐point statistics. Its ranking includes the realizations' variability and their reproduction of the training image.

In InSAR, the simulation of random, spatially correlated signals has mainly been used to estimate the impact of atmospheric and ionospheric delays and of remaining processing errors (e.g., Biggs et al., 2007; Hanssen, 2001; Wright et al., 2004). The eigen decomposition (Lohman & Simons, 2005) and the Cholesky decomposition (Wright et al., 2003, 2004) used in InSAR are classical approaches in statistics to correlate random variables. They rely on building a single covariance matrix between all the variables, on decomposing that covariance matrix, and on multiplying the decomposition by a random vector to obtain correlated samples. However, in a spatial context, each location to simulate corresponds to a random variable, so the size of the covariance matrix makes decomposition‐based methods inefficient for more than a few hundreds locations (Figure 3; Deutsch & Journel, 1992).

This inefficiency triggered the development and large adoption of the SGS in geostatistics. Instead of simulating all locations at once, the SGS proceeds sequentially and exploits two aspects to keep the local covariance matrices as small as possible. First, most spatial fields exhibit a range after which locations become uncorrelated, so only a neighborhood around a given location is worth including in the covariance matrix. Second, the better correlated neighbors screen the less correlated ones, which can be dropped from the covariance matrix (Deutsch & Journel, 1992). While the SGS requires fewer computational resources than decomposition‐based methods, the ranking from an analysis of distance (Tan et al., 2014) shows little influence on the reproduction of the covariance function (Figure 3). And performance becomes key when simulating an artificial interferogram: Our Illapel application includes 22,102,039 cells, so simulating a realization would be impossible using eigen or Cholesky decomposition but only takes a few minutes on a laptop using SGS.

The DS appears more suited to reproduce complex patterns, such as waves often seen in interferograms, while still showing a large variability between realizations (Figure 3). Moreover, it prevents from having to fit a covariance function, which can be a difficult and subjective task. Sentinel‐1 can provide many training images of noise and processing errors when using a small temporal baseline to exclude deformations. Just like SGS, multiple‐point simulations could replace eigen and Cholesky decompositions in InSAR workflows. However, the DS runs slowly compared to its covariance‐based counterpart (Figure 3). Tweaking its parameters helps balance speed and quality of the results (Meerschman et al., 2013), and the DS can be used in an optimization or Monte Carlo setting (Mariethoz et al., 2010b), which could be of major use in InSAR processing (e.g., Elliott et al., 2010; Wright et al., 2003). But an application as large as ours with Illapel would be too demanding to be practical. Fortunately, many studies over the recent years have focused on decreasing the computing cost while improving the quality of the realizations. One solution stands out: simulating patches of cells instead of a single cell at a time (e.g., Hoffimann et al., 2017; Li et al., 2016; Tahmasebi & Sahimi, 2016). It also shows promising applications in a Monte Carlo setting (Pirot et al., 2017; Zahner et al., 2015).

Overall, we believe that the InSAR community can gain from recent developments in geostatistics and machine learning, especially as more and more acquisitions become available. In particular, our approach opens the door for a variety of future uses that distinguish this work from previous simulators (e.g., Biggs et al., 2007; Lee et al., 2012). One such use is a comparison of algorithmic pipelines for InSAR, where this generator can be used to create interferograms with known properties and artifacts as ground truth. Error characterizations on the output can be based on metrics such as the structural similarity index (Wang et al., 2004) between the artificial and real image. With this computer‐aided discovery approach (Pankratius et al., 2016), one can proceed to automatically exchange algorithms and pipeline parameters of the InSAR workflow and ultimately synthesize an algorithmic pipeline in an iterative fashion. Another key application for this generator is a machine learning setting similar to generative adversarial networks (Goodfellow et al., 2014), where the generator is trained to mimic real interferograms in the best possible way, while iteratively measuring error and adjusting the generator's parameter values.

## 5 Conclusions and Perspectives

This article introduces a novel generative approach for artificial InSAR interferograms, which includes parameters for controlling key signal components. The related implementation represents a building block for future InSAR applications and missions like NASA's NISAR, as it allows users to validate and compare different processing workflows and to assess different types of noise contributions. In addition, it opens the door for future applications in more sophisticated machine learning techniques like generative adversarial networks (Goodfellow et al., 2014), which require a generator like this one as a key component whose parameters can be iteratively learned. At that point, when artificial and empirically derived interferograms can no longer be distinguished by a discriminator, the parameters of the generator could give additional insights into the quantitative component contributions for noise. Such information can be then used to select adaptive corrections depending on the situation and to automatically synthesize an improved workflow.

## Appendix A Reference Level and Satellite Position

### A.1

The reference level defines the basic shape of the Earth, from which the topography is defined (Figure 1). This shape can be projected, spherical, or ellipsoidal. The use of those three options is illustrated in the Jupyter notebook /blob/master/geo/insar/simulator/DInSAR_Simulator_Topography.ipynb at (Rude et al., 2017b).

#### A1 Projected Earth

In a projected reference frame, the Earth is shaped as a plane and so is the reference level. Computing coordinates becomes easier.

The satellite's path turns into a line on the projection plane, and we need for each observed point *i* the closest distance to that line, called the ground range $r_{i,1}^{g}$:

(A1) $$\begin{array}{ccc} d_{i,1}^{cross\;ref} & =\kappa (x_{i}^{o}\cos(\beta_{a})-y_{i}^{o}\sin(\beta_{a})),\;\kappa =\left\{\begin{array}{cc} 1,\; & \text{if right looking,}\\ -1,\; & \text{otherwise,} \end{array}\right. & \\ d_{i,1}^{cross} & =c_{i,1}^{cross}-\min_{i}(c_{i,1}^{cross}),\\ r_{i,1}^{g} & =d_{i,1}^{cross}+r_{min,1}^{g}, \end{array}$$

where $d_{i,1}^{cross}$ is the cross‐track distance from the closest observed point to the satellite, *β*_*a*_ is the track angle of the satellite in radians, and $r_{min,1}^{g}$ is the minimal ground range between the first receiver and the closest observed point.

From the ground range, we get the first receiver position for each observed point:

(A2) $$\begin{array}{ccc} x_{i,1}^{r} & =x_{i}^{o}-\kappa \;r_{i,1}^{g}\cos(\beta_{a}), & \\ y_{i,1}^{r} & =y_{i}^{o}+\kappa \;r_{i,1}^{g}\sin(\beta_{a}),\\ z_{i,1}^{r} & =h_{1}^{r}, \end{array}$$

where $h_{1}^{r}$ is the first receiver's elevation from the reference plane.

The second receiver's position is as follows:

(A3) $$\begin{array}{ccc} b_{h} & =b\cos(\alpha ),\;b_{v}=b\sin(\alpha ), & \\ x_{i,2}^{r} & =x_{i,1}^{r}+\kappa \;b_{h}\cos(\beta_{a}),\\ y_{i,2}^{r} & =y_{i,1}^{r}-\kappa \;b_{h}\sin(\beta_{a}),\\ z_{i,2}^{r} & =z_{i,1}^{r}+b_{v}, \end{array}$$

where *b* is the baseline and *alpha* is the angle between the two receivers, *b*_*h*_ is the horizontal baseline, and *b*_*v*_ is the vertical baseline. Equation (2) provides the slant range of each receiver.

#### A2 Spherical Earth

Projections simplify calculations but lead to noticeable approximations over large areas and remove Earth's shape from the interferograms. A first solution considers the Earth as a sphere so the reference level as a sphere. Instead of working with projected coordinates in meters, we start from a spherical coordinate system: the longitude *λ* and the geographic latitude *φ* in radians.

On a curved surface, a geodesic is analogous to a straight line on a plane: It represents the shortest path between two points. On a sphere, a geodesic corresponds to a great circle. Coordinates, distances, and bearings can be retrieved based on spherical trigonometry, which gives exact results for a sphere.

To compute the ground range, we assume the satellite path follows a great circle, and we compute the shortest distance between each observed point and that great circle. We know the bearing of the great circle but not its location. We first compute the shortest distance $d_{i}^{cross\;ref}$ to a great circle with the same bearing but passing through a point selected as reference $\left(\lambda_{ref}^{o},\varphi_{ref}^{o}\right)$, which in our implementation is the point of coordinate (0, 0) in the domain's grid but can be any point. The distance $d_{i}^{ref}$ and bearing $\theta_{i}^{ref}$ between all the observed points $\left(\lambda_{i}^{o},\varphi_{i}^{o}\right)$ and the reference point become

(A4) $$\begin{array}{ccc} \Delta \lambda & =\lambda_{ref}^{o}-\lambda_{i}^{o}, & \\ \Delta \varphi & =\varphi_{ref}^{o}-\varphi_{i}^{o},\\ d_{i}^{ref} & =2\;r_{e}\arcsin\left(\sqrt{\sin^{2}\left(\frac{\Delta \varphi }{2}\right)+\cos\left(\varphi_{ref}^{o}\right)\cos\left(\varphi_{i}^{o}\right)\sin^{2}\left(\frac{\Delta \lambda }{2}\right)}\right),\\ \theta_{i}^{ref} & =\text{arctan2}\left(\sin(\Delta \lambda )\cos(\varphi_{i}^{o}),\cos(\varphi_{ref}^{o})\sin(\varphi_{i}^{o})-\sin\left(\varphi_{ref}^{o}\right)\cos\left(\varphi_{i}^{o}\right)\cos\left(\Delta \lambda \right)\right),\\ d_{i,1}^{cross\;ref} & =\kappa \;r_{e}\arcsin\left(\sin\left(\frac{d_{i}^{ref}}{r_{e}}\right)\sin\left(\theta_{i}^{ref}-\beta_{a}\right)\right),\;\kappa =\left\{\begin{array}{ll} 1, & \text{if right looking},\\ -1, & \text{otherwise,} \end{array}\right. \end{array}$$

where *r*_*e*_ is the Earth's radius, 6,371,008.8 m. The ground range $r_{i,1}^{g}$ is then

(A5) $$\begin{array}{ccc} d_{i,1}^{cross} & =d_{i,1}^{cross\;ref}-\min_{i}(d_{i,1}^{cross\;ref}), & \\ r_{i,1}^{g} & =d_{i,1}^{cross}+r_{min,1}^{g}, \end{array}$$

where *β*_*a*_ is the track angle of the satellite in radians.

From the ground range, we compute the longitude $\lambda_{i,1}^{r}$ and latitude $\varphi_{i,1}^{r}$ of the first receiver:

(A6) $$\begin{array}{ccc} \varphi_{i,1}^{r} & =\arcsin\left(\sin(\varphi_{i}^{o})\cos\left(\frac{r_{i,1}^{g}}{r_{e}}\right)+\cos(\varphi_{i}^{o})\sin\left(\frac{r_{i,1}^{g}}{r_{e}}\right)\cos(\beta_{c}+\pi )\right), & \\ \lambda_{i,1}^{r} & =\lambda_{i}^{o}+\text{arctan2}\left(\sin(\beta_{c}+\pi )\sin\left(\frac{r_{i,1}^{g}}{r_{e}}\right)\cos(\varphi_{i}^{o}),\cos\left(\frac{r_{i,1}^{g}}{r_{e}}\right)-\sin(\varphi_{i}^{o})\sin(\varphi_{i,1}^{r})\right), \end{array}$$

where *β*_*c*_ is the azimuth of the cross‐track direction in radians.

To use equation (2), we convert the satellite's coordinates $\left(\lambda_{i,1}^{r},\varphi_{i,1}^{r},h_{1}^{r}\right)$ into a Cartesian reference frame in meters with the center of the Earth as origin:

(A7) $$\begin{array}{ccc} x_{i,1}^{r} & =(r_{e}+h_{1}^{r})\cos(\varphi_{i,1}^{r})\cos(\lambda_{i,1}^{r}), & \\ y_{i,1}^{r} & =(r_{e}+h_{1}^{r})\cos(\varphi_{i,1}^{r})\sin(\lambda_{i,1}^{r}),\\ z_{i,1}^{r} & =(r_{e}+h_{1}^{r})\sin(\varphi_{i,1}^{r}), \end{array}$$

where $h_{1}^{r}$ is the first receiver's elevation from the reference plane.

Obtaining the second receiver's coordinates relies on the same equations:

(A8) $$\begin{array}{ccc} b_{h} & =b\cos(\alpha ), & \\ \varphi_{i,2}^{r} & =\arcsin\left(\sin(\varphi_{i,1}^{r})\cos\left(\frac{b_{h}}{r_{e}}\right)+\cos(\varphi_{i,1}^{r})\sin\left(\frac{b_{h}}{r_{e}}\right)\cos(\beta_{c})\right),\\ \lambda_{i,2}^{r} & =\lambda_{i,1}^{r}+\text{arctan2}\left(\sin(\beta_{c})\sin\left(\frac{b_{h}}{r_{e}}\right)\cos(\varphi_{i,1}^{r}),\cos\left(\frac{b_{h}}{r_{e}}\right)-\sin(\varphi_{i,1}^{r})\sin(\varphi_{i,2}^{r})\right), \end{array}$$

where *b* is the baseline, *α* the angle between the two receivers, and *b*_*h*_ is the horizontal baseline.

(A9) $$\begin{array}{ccc} b_{v} & =b\sin(\alpha ), & \\ h_{2}^{r} & =h_{1}^{r}+b_{v},\\ x_{i,2}^{r} & =(r_{e}+h_{2}^{r})\cos(\varphi_{i,2}^{r})\cos(\lambda_{i,2}^{r}),\\ y_{i,2}^{r} & =(r_{e}+h_{2}^{r})\cos(\varphi_{i,2}^{r})\sin(\lambda_{i,2}^{r}),\\ z_{i,2}^{r} & =(r_{e}+h_{2}^{r})\sin(\varphi_{i,2}^{r}), \end{array}$$

where *b*_*v*_ is the vertical baseline and $h_{2}^{r}$ is the second receiver's elevation from the reference plane. To get the slant range for each receiver (equation 2), we need to convert the coordinates of the observed points into the same reference frame:

(A10) $$\begin{array}{ccc} x_{i}^{o} & =(r_{e}+h_{i}^{o})\cos(\varphi_{i}^{o})\cos(\lambda_{i}^{o}), & \\ y_{i}^{o} & =(r_{e}+h_{i}^{o})\cos(\varphi_{i}^{o})\sin(\lambda_{i}^{o}),\\ z_{i}^{o} & =(r_{e}+h_{i}^{o})\sin(\varphi_{i}^{o}), \end{array}$$

where $\lambda_{i}^{o}$, $\varphi_{i}^{o}$, and $h_{i}^{o}$ are the longitude, latitude, and elevation, respectively, from the reference plane (i.e., the topography) of the observed point *i*.

#### A3 Ellipsoidal Earth

Earth's shape is more an oblate ellipsoid than a sphere, and an ellipsoidal reference level provides a better approximation. Again, we start from the longitude *λ* and the geographic latitude *φ* in radians.

The definition of a geodesic still holds for an ellipsoid, but it is not a great circle anymore. Thus, we cannot directly rely on spherical trigonometry but on optimization methods to approximate coordinates, distances, and bearings. Some methods can reach an accuracy of less than a millimeter, high enough for most cases.

We assume that the satellite's path follows a geodesic, so computing the ground range is equivalent to computing the shortest distance between all the observed points and that geodesic. We know the bearing of the geodesic but not its location. We first compute the shortest distance $d_{i}^{cross\;ref}$ to a geodesic with the same bearing but passing through a point selected as reference $\left(\lambda_{ref}^{o},\varphi_{ref}^{o}\right)$, which again is the point of coordinate (0, 0) in the domain's grid. Few studies have been dedicated to computing the shortest distance to a geodesic on an oblate ellipsoid. We rely on the method developed by Baselga and Martínez‐Llario (2017), using the WGS84 ellipsoid (*a* = 6,378,137 m and *f* = $\frac{1}{298.257223563}$). Once we have $d_{i}^{cross\;ref}$, the ground range $r_{i,1}^{g}$ becomes

(A11) $$\begin{array}{ccc} d_{i,1}^{cross} & =d_{i,1}^{cross\;ref}-\min_{i}(d_{i,1}^{cross\;ref}), & \\ r_{i,1}^{g} & =d_{i,1}^{cross}+r_{min,1}^{g}. \end{array}$$

From the ground range, we compute the longitude $\lambda_{i,1}^{r}$ and latitude $\varphi_{i,1}^{r}$ of the first receiver using Vincenty's direct formula (Vincenty, 1975). This formula computes the longitude and latitude of a point knowing an origin point and the distance and bearing between the two points. It is also one of the basic component of Baselga and Martínez‐Llario's (2017) method. Here, the origin point is an observed point *i* of coordinates $\left(\lambda_{i}^{o},\varphi_{i}^{o}\right)$, the distance is the ground range, and the bearing is *β*_*c*_+*π*, with *β*_*c*_ the azimuth of the cross‐track direction in radians.

To use equation (2), we convert the satellite's coordinates $\left(\lambda_{i,1}^{r},\varphi_{i,1}^{r},h_{1}^{r}\right)$ into a Cartesian reference frame in meters with the center of the Earth as origin:

(A12) $$\begin{array}{ccc} b & =a(1-f), & \\ e^{2} & =1-{\left(\frac{b}{a}\right)}^{2},\\ r_{v} & =\frac{a}{\sqrt{1-e^{2}\sin^{2}(\varphi_{i,1}^{r})}},\\ x_{i,1}^{r} & =(r_{v}+h_{1}^{r})\cos(\varphi_{i,1}^{r})\cos(\lambda_{i,1}^{r}),\\ y_{i,1}^{r} & =(r_{v}+h_{1}^{r})\cos(\varphi_{i,1}^{r})\sin(\lambda_{i,1}^{r}),\\ z_{i,1}^{r} & =(r_{v}(1-e^{2})+h_{1}^{r})\sin(\varphi_{i,1}^{r}), \end{array}$$

where *a* and *b* are the semimajor and semiminor axes of the oblate ellipsoid, *f* and *e* are the flattening and eccentricity of the ellipsoid, *r*_*v*_ is the radius in the prime vertical, and $h_{1}^{r}$ is the first receiver's elevation from the ellipsoid.

The same operations compute the coordinates of the second receiver. The longitude $\lambda_{i,2}^{r}$ and latitude $\varphi_{i,2}^{r}$ result from Baselga and Martínez‐Llario's (2017) method, where the origin point is the first receiver location $\left(\lambda_{i,1}^{r},\varphi_{i,1}^{r}\right)$, the distance is the horizontal baseline $b_{h}=b\cos(\alpha )$, and the bearing is *β*_*c*_. Then,

(A13) $$\begin{array}{ccc} b_{v} & =b\sin(\alpha ), & \\ h_{2}^{r} & =h_{1}^{r}+b_{v},\\ r_{v} & =\frac{a}{\sqrt{1-e^{2}\sin^{2}(\varphi_{i,2}^{r})}},\\ x_{i,2}^{r} & =(r_{v}+h_{2}^{r})\cos(\varphi_{i,2}^{r})\cos(\lambda_{i,2}^{r}),\\ y_{i,2}^{r} & =(r_{v}+h_{2}^{r})\cos(\varphi_{i,2}^{r})\sin(\lambda_{i,2}^{r}),\\ z_{i,2}^{r} & =(r_{v}(1-e^{2})+h_{2}^{r})\sin(\varphi_{i,2}^{r}), \end{array}$$

where *b* is the baseline, *α* the angle between the two receivers, *b*_*h*_ is the horizontal baseline, and $h_{2}^{r}$ is the second receiver's elevation from the ellipsoid. To get the slant range for each receiver (equation 2), we need to convert the coordinates of the observed points into the same reference frame:

(A14) $$\begin{array}{ccc} r_{v} & =\frac{a}{\sqrt{1-e^{2}\sin^{2}(\varphi_{i}^{o})}}, & \\ x_{i}^{o} & =(r_{v}+h_{i}^{o})\cos(\varphi_{i}^{o})\cos(\lambda_{i}^{o}),\\ y_{i}^{o} & =(r_{v}+h_{i}^{o})\cos(\varphi_{i}^{o})\sin(\lambda_{i}^{o}),\\ z_{i}^{o} & =(r_{v}(1-e^{2})+h_{i}^{o})\sin(\varphi_{i}^{o}), \end{array}$$

where $\lambda_{i}^{o}$, $\varphi_{i}^{o}$, and $h_{i}^{o}$ are the longitude, latitude, and elevation, respectively, from the ellipsoid of the observed point *i*.
